# What influences patient decisions when selecting an obesity treatment?

**DOI:** 10.1016/j.obpill.2024.100123

**Published:** 2024-08-08

**Authors:** Hilary C. Craig, David Walley, Carel W. le Roux

**Affiliations:** a Diabetes Complications Research Centre, Conway Institute of Biomedical and Biomolecular Research, University College Dublin, Dublin 4, Ireland; b UCD School of Medicine, University College Dublin, Dublin 4, Ireland

## Abstract

**Objective:**

The objectives of this study were to understand patient preferences for obesity treatments, to describe how patients choose treatment options, and what factors influence their decisions.

**Methods:**

This participatory action research used purposeful sampling to recruit 10 patients with complications of obesity. Photovoice was used as the qualitative research methodology. Recruitment took place in specialist clinics for metabolic dysfunction-associated steatotic liver disease, diabetes mellitus, hypertension, and chronic kidney disease. Two males and eight females aged 18–75 years, with a BMI greater than 35 kg/m^2^ were recruited. Participants watched a 60-min ​video explaining nutritional, pharmacological, and surgical therapies in equipoise. Data was collected using photographs with a disposal camera followed by one-to-one semi-structured interviews. Afterward, this analysis utilised reflective thematic analysis.

**Results:**

Five main themes were identified that influenced patients' decisions when selecting an obesity treatment: 1] Accessibility issues, 2] Polypharmacy, 3] Fears around future health 4] Lack of Support 5] Information Mismanagement.

**Conclusion:**

The themes identified in this study represent the patients’ voices for those living with obesity complications and what influences their decisions on treatment options. The findings underscore the need for a holistic and patient-centred approach to the management of obesity and its associated complications. Patient-centred care including knowledge, health literacy, support, and participation is essential to providing effective care for patients with obesity to make decisions between treatment options.

## Graphical abstract

**Figure:**
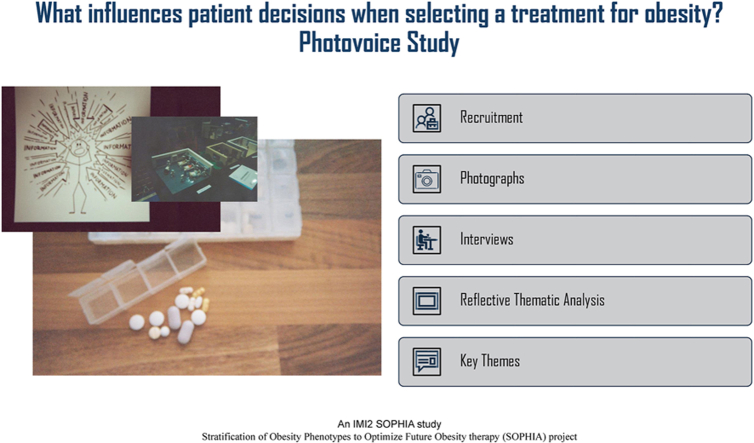

## 1 Introduction

**Fig. 1:**
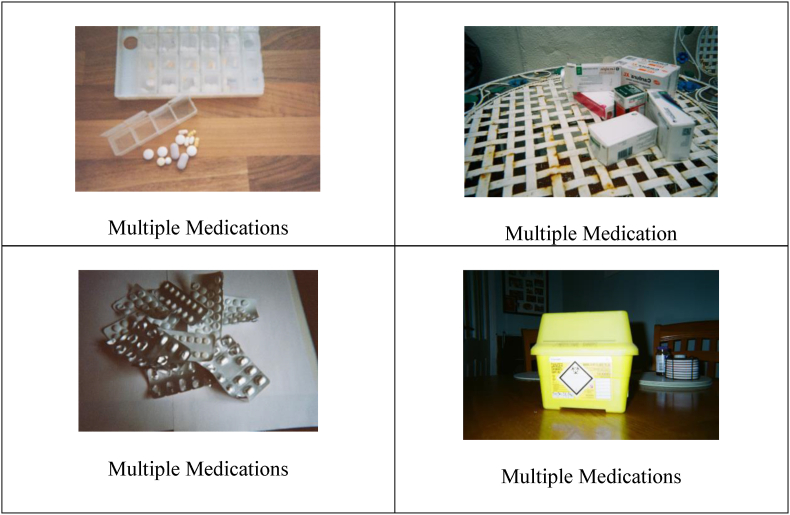
Polypharmacy.

**Fig. 2:**
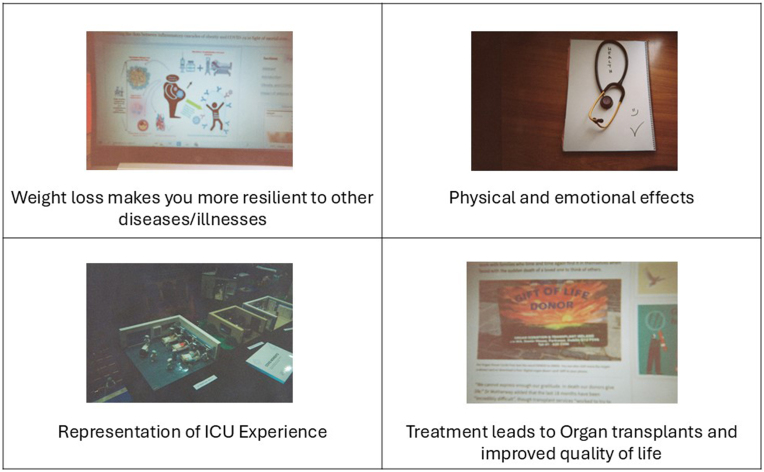
Fears around future health.

**Fig. 3:**
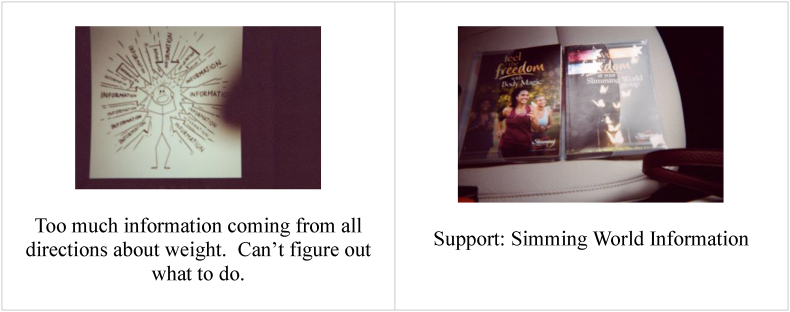
Information mismanagement.

**Fig. 4:**
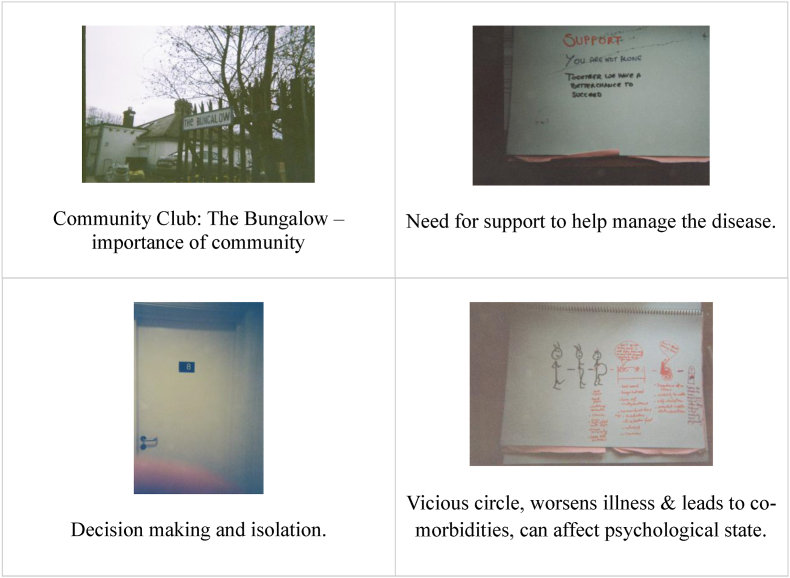
Support.

The Obesity Medicine Association defines obesity as a serious, chronic, progressive, relapsing, and treatable multi-factorial, neurobehavioral disease, wherein an increase in body fat promotes adipose tissue dysfunction and abnormal fat mass physical forces, resulting in adverse metabolic, biomechanical, and psychosocial health consequences [1]. Patients living with obesity face both increased morbidity and mortality [2,3], while also being limited when performing day-to-day tasks, such as climbing stairs. Obesity is associated with more than 220 different complications such as type 2 diabetes mellitus (T2D), metabolic dysfunction-associated steatotic liver disease (MASLD), and chronic kidney disease (CKD), which also limit a patient’s abilities [4]. Nutritional, exercise, pharmacological, and surgical therapies are evidence-based obesity treatments that successfully treat the disease [5], but the level of weight loss can vary substantially between and within each option [6,7]. The importance of the patient voice has become increasingly recognised by healthcare professionals [8]. Not only does patient involvement uphold the ethical principle of autonomy, but it also improves patient outcomes. In this study, our objective was to understand the voices of patients living with obesity complications regarding the factors involved in selecting between treatment options for obesity (see Table 1, Table 2, Fig. 1, Fig. 2, Fig. 3, Fig. 4).

**Table 1:** Characteristics of participants.

Age	Sex
18–75 years	Two Males
	Eight Females
**Health Complications**	
60 % of participants had MASLD	
40 % of participants had T2DM	
40 % of participants had hypertension	
20 % of participants had CKD

**Table 2:** Themes.

Accessibility Issues	Polypharmacy	Fears around Future Health	Lack of Support	Information Mismanagement

## 2 Methods

This qualitative study used Photovoice, a form of community-based participatory action research that facilitates understanding of the patient experience through visual representation [9]. The Photovoice framework encouraged thorough discussion and reflection through the use of photographs taken by the participant. The advantages of Photovoice are that it is interactive, supports qualitative discussion, and can be empowering for participants. The disadvantages of Photovoice are that it can take longer than one-to-one interviews, and recruitment can be challenging as it involves more tangible engagement from participants. The interview questions were discussed with the research team and developed using previous research as well as experiences from the Stratification of Obesity Phenotypes to Optimize Future Obesity Therapy (SOPHIA) project. None of the research team had any preferences for treatment options for obesity or any themes that may have emerged to support the choices of patients.

In summary, Photovoice is a collaborative approach that allows participants to deepen their experiences by representing their lived experiences through images. The process includes a discussion of patient images to gain an understanding of common issues, context, and solutions [14]. This provided a deeper perspective on their experiences, views, and their future desires based on treatment options. Sharing photographs and feedback to the researcher enabled participants to reflect on their current situation and to see improvements they may make. It is a useful way to reveal knowledge gaps and improve the quality and efficiency of care. The use of photographs enables the participants to demonstrate their concerns; an example is how the emotional and physical impact of their conditions governs how they interact and make decisions on what treatment for obesity to select.

### 2.1 Ethics and consent

This study was approved by the University College Dublin Human Research Ethics Committee (LS-21-58-LeRoux) and conducted according to the Declaration of Helsinki. Participants provided written informed consent before the Photovoice methodology.

### 2.2 Recruitment

Ten patients with obesity complications were recruited for this study. To ensure consent for this study all details relating to it were provided. This included providing a patient information leaflet explaining the study, how the data will be used, how the data will be stored, and confidentiality information. The participants were informed of the benefits and risks of being part of the study and options should they change their minds. Participants were then invited to consent to Photovoice after they had a chance to think about it. None of these patients had a formal diagnosis of obesity or had they been verbally diagnosed with obesity. Participants were not attending obesity-specific clinics but rather multiple clinics for complications driven by obesity. Recruitment took place in clinics for MASLD, T2DM, hypertension, and CKD within two large general hospitals, St James’s Hospital and St Vincent’s Hospital in Dublin, Ireland. Two male patients and eight female patients were included in the study. The age of participants ranged from 18 to 75 years. Obesity was assessed using BMI and all patients’ BMIs were greater than 35 kg/m^2^.

### 2.3 Camera distribution

Disposable cameras were used by participants to take photographs. A package containing a disposable camera, instructions, an information leaflet, and a self-addressed stamped envelope (SASE) was sent to each participant. Participants were instructed to take 25 photographs overall, which included:

− 10 photographs to represent why they would select one obesity treatment above another based on their concerns about living with obesity complications.
− 10 photographs to represent why they would select one obesity treatment above another based on their hopes and desires to engage with treatment.
- Five photographs to represent why they would select one obesity treatment above another based on their experience of living with obesity during the COVID-19 pandemic

Patients submitted a range of 12–27 photographs. Participants had two weeks to take the photographs using the disposable camera. Thereafter, the participants returned the disposable camera to the research centre. Upon receipt, the film was developed, and photographs were arranged on a slideshow presentation where each photograph was assigned a number.

### 2.4 Interview

Participants were asked to watch a 60-min ​video explaining nutritional and exercise, pharmacological, and surgical therapies in equipoise. Due to public health restrictions for the COVID-19 pandemic, interviews with participants were conducted virtually using the video chat platform ‘Zoom’ where the researcher would describe the pictures they received from the participants who then expanded on what the meaning of it was to them. The interview was led by the first author who served as the primary contact for participants. The interview began with general questions regarding the patient’s choices of treatment based on their experience of living with obesity. Afterward, both the interviewer and participant observed the slideshow presentation of the photographs that the patient took using the disposable camera. At first, the patient discussed the meaning of the photo, which was then followed by clarifying questions from the interviewer. No leading questions were asked to avoid introducing bias. At the end of the interview, the participant was asked by the interviewer to select three to five photographs from the slideshow that best represented the three aforementioned themes. Each participant’s photographs were collated for thematic analysis later. The interviews ranged from 25 to 60 min. Where possible, interviews were transcribed using built-in transcription software. This transcript was then reviewed in detail by a member of the research team resulting in a verbatim transcript.

### 2.5 Thematic analysis

Thematic analysis was conducted utilising Clarke and Braun’s (2013) six-step data analysis process which involves familiarisation of data, generation of codes, combining codes into themes, reviewing themes, determining the significance of themes, and reporting the findings [10]. This analysis was conducted using a total of fifty photographs selected by each participant as well as transcripts from the ten interviews. The coding framework was based on previous research on the topic and the interview transcripts. Transcripts were anonymised and added to MAXQDA 2022 plus software to aid the coding of the data. The photo data was interpreted alongside the related transcript to ensure analysis was based on the patient’s interpretation of the image. A total of 155 photographs were received representing five main themes. Themes were identified, refined, and agreed upon by all authors.

## 3 Results

Fifty percent of participants chose nutritional and exercise therapies. This was driven by their view that it was a more sustainable option, improved health, and helped with psychological well-being. Participants also expressed their fears of polypharmacy and the potential side effects of medications as a reason for opting for their choice. Thirty percent chose medication. This was driven by the participants’ view that it helped with their current complications of obesity, their concerns for the future, and their need to improve their health. For some, it was considered cheaper than surgery. Twenty percent chose surgery as their treatment choice because it was a one-step process compared to being on medication for life. Surgery was also thought to improve quality of life, and it was a more immediate solution if there was a life decision, such as planning to start a family. Participant voices were further validated through the selected photographs they took, which represented their experiences, the influences around their choices, and their treatment options. Five main themes were identified: 1] Accessibility issues, 2] Polypharmacy, 3] Fears around future health 4] Lack of Support 5] Information Mismanagement.

Access and cost were some of the themes identified, particularly around the cost of medications, which was perceived as a barrier to managing their care. Participants raised concerns about long waiting lists for surgery and sometimes being advised by their healthcare professionals (HCPs) to go overseas for private surgical treatments. This would be costly but the public waiting list meant they would be waiting several years before receiving treatment. Patients who selected the nutritional therapy and exercise therapy options mentioned the importance and challenges of accessing support in trying to manage their disease. Patients who selected pharmacotherapy commented that the new generation of medication offers hope, but the cost was a challenge. Patients who selected surgery explained they would have the operation tomorrow if it was offered to them.

## 4 Polypharmacy

One of the main factors emphasised by participants that led them to their treatment choices was deteriorating health and having to take multiple medications for a variety of conditions. Polypharmacy influenced participants as many were reluctant to take additional medications. Participants were concerned about side effects and having to take more medications unsure of what the outcome may be for them. Patients who selected nutritional therapies and exercise therapies mentioned that they did not want to be on multiple medications for life. Patients who selected pharmacotherapy commented that they were in a dilemma because they needed to take several medications to treat some of the complications of obesity, but they were hoping medications for obesity may reduce the need for other medications for the complications of obesity. Patients who selected surgery explained that moving from needing several medications to not needing them for the complications of obesity would be a huge improvement for them.

### 4.1 Fears around future health

The physical impact of obesity affected their quality of life as decisions were influenced by illness or a lack of mobility. Participants described the effort it takes to actively participate in society and how planning becomes a vital part of engaging in any social situation and can prevent making plans.

Obesity can be the cause of a series of associated complications [11]. The physical impact of obesity had a considerable impact on how participants engaged in activities with family and friends. Poor mobility, joint pain, and lack of energy impact health-related quality of life. This was highlighted through the participants’ experiences during the COVID-19 pandemic. Fear of life was a sharp reality for many participants and concerns from family and friends about what would happen if the participant got infected caused constant anxiety. Participants expressed their fears of losing a limb because of the complications of obesity. The increasing challenges of obesity have increased interest in health and quality of life outcomes [12]. Sharafi et al. found that obesity was associated with the incidence of anxiety and depression and indeed that depression and anxiety were found to be risk factors for developing obesity indicating an association between psychological states and obesity [13]. The emotional as well as the physical impact of obesity affected participants’ quality of life. Feelings of shame, low self-esteem, isolation, and concerns about the negative health consequences were some of the concerns highlighted by participants. Participants outlined how emotional factors such as depression, anxiety, and future worries influence decision-making regarding weight-loss strategies and future treatment options. Patients who selected lifestyle options mentioned if their health deteriorated, they would choose other options like surgery or medication, but that they hoped lifestyle changes may be enough to maintain their health. Patients who selected pharmacotherapy commented that they have to face where they are because it was becoming the frontier between the life they would like to live and the life they have to live. Thus taking medication was a price they would be prepared to pay. Patients who selected surgery explained they did not want their health to get worse or that they might lose a limb. Moreover, they found it challenging to deal with obesity complications in social settings as people feel it is okay to comment on their conditions. Thus, having a definitive treatment to ensure better future health was worth it.

### 4.2 Information Mismanagement

Participants described their confusion about obtaining the most appropriate information about treatment options. Information comes from multiple sources and there is either too much or there is too little, so it is difficult for them to establish who or what to rely on. Participants did not understand all the information coming to them and described how difficult it is to judge or decide what treatment was right for them unless they fully understand all the options. When they were looking at how to get a diagnosis and get treatment, they were seeking the tools to know how they take care of themselves and what choice would be right for them. Participants experienced diverse levels of information from HCPs which can be confusing. Patients who selected lifestyle options mentioned that information was power but there is so much coming from all directions it was hard to know what the correct information was, however, they felt lifestyle options were improving and hence worth sticking with. Patients who selected pharmacotherapy commented that their HCP did not have enough information on new medications, and they were left trying to find information on different medications, but that the information they have been able to access has encouraged them to consider medications. Patients who selected surgery explained there was a lack of information on surgical options locally and indeed the suggestion of going overseas to have the surgery was mentioned.

### 4.3 lack of support

Support from family, friends, and their HCPs was considered of significant importance to the participants in deciding on treatment options. Participants found community support helpful in terms of community group activities which supported their efforts and improved their social interactions.

In addition, participants recognised the importance of their physicians’ perspectives and were deeply and negatively affected in cases where the HCP dismissed their concerns and fears and did not provide support, knowledge, or solutions. Participants felt they were not heard and were isolated in managing their conditions and isolated in having to make decisions about which treatment options would be the best option for them. As one participant described it, they were confused as to which door to choose. The lack of support and information has led to unsupported self-care and decision-making, leading patients to do this in isolation. Patients who selected lifestyle options mentioned the importance of support from family and the community but also from someone who knows what they are talking about and who has experience in helping people, is vital. Thus, they felt although challenging there were better options within the healthcare system but also by commercial providers of nutrition and exercise therapies. Patients who selected pharmacotherapy commented that isolation, lack of socialising, and being able to have a normal life affect their choice, especially the feeling the doors are being shut at every turn. Thus, they thought that medications may improve support if provided within the healthcare system as part of usual care. Patients who selected surgery explained the importance of mental health and how it can affect your decisions, your self-confidence, and how you live your life. They thought that because surgery was provided by specialist hospitals it may also be associated with better support

## 5 Discussion

When offered all the obesity treatment options in equipoise, half of patients with obesity complications selected nutritional and exercise therapies, 30 % wanted pharmacotherapy, and 20 % selected surgical therapies. Five main themes that influenced patients’ decisions when selecting an obesity treatment were: 1] Accessibility issues, 2] Polypharmacy, 3] Fears around future health 4] Lack of Support 5] Information Mismanagement.

The requirement to be on multiple medications daily was a concern along with deteriorating mobility and function impacted decision-making on treatment options. As this research was conducted during and just after the COVID-19 pandemic the impact of public health restrictions had a significant effect on some participant's selected treatment modalities. Social isolation and fear were the experiences of participants whose family and friends, while trying to be supportive, were emphasising they were at risk. As Farrell et al. outlined people with obesity reported feeling ‘othered’ by their ‘at risk’ categorisation and indeed the public health messaging related to obesity made people feel segregated and punished by society [15]. This is consistent with the experiences of participants in this study who specifically described feeling lonely and how this impacted their decisions regarding obesity treatments.

Although many photographs described the participants’ experiences, the role of cost and access to treatment was one of the main findings impacting treatment choice. Obesity medication was perceived as helpful but also motivated participants to do as much as possible before resorting to medication. Fear was a common theme around health complications and participants talked about their physical challenges and the fear of what lies ahead for them if they select one treatment above another.

Knowledge and information were central to participants’ treatment decisions, especially the need to improve their health literacy as they attempt to navigate the often overburdening amount of information they need to process about their health and treatment options. The importance of receiving clear and meaningful information and feedback from their HCPs was critical for participants and their treatment decisions. They identified that information, feedback, and involvement with their HCPs were also key to enabling them to consider ways to support their decision-making process regarding treatment options. Greater knowledge about obesity and obesity treatments among HCPs is needed. Wynn et al. found that many HCPs have low levels of knowledge about obesity and obesity treatments. They recommended educational strategies aimed at HCPs to improve the care provided to patients with obesity and its associated complications [16]. The benefit of receiving support was another theme that was identified particularly from families, friends, HCPs, and the community as a whole by creating a culture of support. Participants expressed that they managed their conditions more effectively when they got support from all sides of the community.

Patient preferences were influenced by elements such as patients’ beliefs, values, expectations, and health goals. They included the way patients manage the benefits and limitations of treatment options. These elements are complex, as people may make decisions from different perspectives including emotional and social issues. The participants in this study described the need for more concise and clear information, better communication, and access to care and support. It is difficult for them to make informed choices or to state their preferences for a particular treatment. All of these elements are components of patient-centred care which can improve outcomes but also assist patients in deciding on treatment choices.

The themes identified outline the essential need for patient-centred care to assist decision-making regarding obesity treatments. John Fastenau et al. outlined the aspects of patient-centred care comprising eight dimensions i.e. respect for patients' preferences, physical comfort, the coordination of care, emotional support, access to care, the continuity of care, the provision of information and education, and the involvement of family and friends [17,18]. These dimensions were being sought by the participants to manage their chronic conditions and improve their quality of life. Participants treatment decision choices were primarily related to their quality-of-life issues, health benefits, and sustainability. Their decisions were made regarding how each choice fit the participants' lifestyle, well-being, and health benefits to improve existing conditions. Recognising the importance patients place on improving their health and quality of life to achieve sustainable health benefits can inform how we approach and support patients in their choices regarding obesity treatments.

### 5.1 Strengths and limitations

The strength of this study is the active engagement with the participants in the sharing of their experiences. The participants had an opportunity to reflect on these experiences in a meaningful way through their photographs. Several mentioned that participating has made them think about what they can do or change to make things better for themselves which is consistent in other studies [19]. This participatory action research inquiry enabled participants to represent their voice particularly as they were requested to identify three to five photographs that were of the greatest importance to them to represent their views. Barry et al. (2021) outlined how people participate in photovoice with great uncertainty and this requires sensitivity and openness to new ideas [20]. The limitation of this method is that it requires more involvement and time on the part of the participant, and this can be a challenge when recruiting participants, but also for participants to complete all the research tasks. Additional limitations included that participants sometimes submitted fewer photographs than requested, albeit the quality of the interviews was unaffected. The number of participants recruited was relatively small but was more than adequate to achieve saturation of themes to describe how patients select treatment options for obesity. An inherent limitation of all qualitative research is that the findings only pertain to those studied, albeit the emerging themes can be used in subsequent qualitative and quantitative research to better understand the research question.

## 6 Conclusion

Factors that influenced participants’ treatment choice included clear information, access, support, polypharmacy, and fears for the future i.e. physical health limitations. Their choices for nutritional and exercise therapies were driven primarily by sustainability, health gains, and wellbeing, while drivers for pharmacotherapies were health gains, and for surgery were to improve their quality of life and/or enable them to make significant life choices. The findings underscore the need for a holistic and patient-centred approach to the management of obesity and its associated complications. Patient-centred care including knowledge, health literacy, support, and participation is essential to providing effective care for patients with obesity to make decisions between treatment options.

Key takeaways clinical messages.

• Factors that influenced participants’ treatment choice included clear information, access, support, polypharmacy, and fears for the future i.e. physical health limitations
• Choices for nutritional and exercise therapies were driven primarily by sustainability, health gains, and wellbeing, while drivers for pharmacotherapies were health gains, and for surgery were to improve their quality of life and/or enable them to make significant life choices.
• The findings underscore the need for a holistic and patient-centred approach including knowledge, health literacy, and support, to the management of obesity and its associated complications.

## Ethics and consent

This study was approved by the University College Dublin Human Research Ethics Committee (LS-21-58-LeRoux) and conducted according to the Declaration of Helsinki. Participants provided written informed consent before the Photovoice methodology. To ensure consent for this study all details relating to it were provided. This included providing a patient information leaflet explaining the study, and how the data will be used, and stored. The participants were informed of the benefits and risks of being part of the study and options should they change their minds. Participants were then invited to consent to Photovoice.

## Funding

This research was funded by the 10.13039/501100002081Irish Research Council, 10.13039/501100001602Science Foundation Ireland, Health Research Board, Anabio, and the Stratification of Obesity Phenotypes to Optimize Future Obesity Therapy (SOPHIA) project.

## Declaration of Artificial Intelligence (AI) and AI-assisted technologies

No AI was used in the development of this manuscript.

## Contact information

Hilary C. Craig, Diabetes Complications Research Centre, UCD Conway Institute of Biomedical and Biomolecular Research, School of Medicine, University College Dublin, Belfield, Dublin 4, Ireland, Hilary.Craig1@ucdconnect.ie,

Hilary Craig: Conceptualisation, formal analysis, funding acquisition, methodology, Literature search, figures, study design, data collection, data analysis, data interpretation, writing, review, and editing. Prof Carel le Roux: Conceptualisation, formal analysis, funding acquisition, methodology, supervision, validation, writing review, and editing. David Walley, Conceptualisation, data collection, writing, review and editing. SOPHIA (the Stratification of Obesity Phenotypes to Optimize Future Obesity Therapy (SOPHIA) project (www.imisophia.eu)) Review Committee: Formal analysis, review, and editing.

## Declaration of competing interest

The authors declare the following financial interests/personal relationships which may be considered as potential competing interests:Hilary C. Craig, reports full funding of PhD tuition fees from the Stratification of Obesity Phenotypes to Optimize Future Obesity Therapy (SOPHIA) project (www.imisophia.eu). reports support provided by University College Dublin Prof Carel le Roux reports a relationship with 10.13039/501100001631University College Dublin that includes: board membership, consulting or advisory, equity or stocks, speaking and lecture fees, and travel reimbursement. Hilary C. Craig, funding of PhD tuition fees from the Stratification of Obesity Phenotypes to Optimize Future Obesity Therapy (SOPHIA) project (www.imisophia.eu). David Walley – no conflict of interest. Carel W. le Roux: Consulting fees/Honoria/Support for meetings: NovoNordisk, Eli Lilly, Johnson & Johnson, Boehringer Ingelheim, GI Dynamics, Herbalife. Leadership/fiduciary role in Board: Irish Society for Nutrition and Metabolism (unpaid). Stock Options: Keyron Previous Chief medical officer and Director of the Medical Device Division of Keyron in 2011. Both of these were unremunerated positions. Previous investor in Keyron, which develops endoscopically implantable medical devices intended to mimic the surgical procedures of sleeve gastrectomy and gastric bypass. He continues to provide scientific advice to Keyron for no remuneration If there are other authors, they declare that they have no known competing financial interests or personal relationships that could have appeared to influence the work reported in this paper.
